# Visual Outcomes and Prognostic Factors after Pars Plana Vitrectomy for Traumatic Endophthalmitis

**DOI:** 10.1155/2017/5851318

**Published:** 2017-01-26

**Authors:** Tao Jiang, Jing Jiang, Renping Wang, Jianlin Lei, Yang Zhou

**Affiliations:** ^1^Department of Ophthalmology, The Affiliated Hospital of Qingdao University, Qingdao, Shandong Province 266003, China; ^2^The Infectious Diseases Department, Eastern Branch of the Affiliated Hospital of Qingdao University, Qingdao, Shandong Province 266061, China; ^3^Health Examination Center, The Affiliated Hospital of Qingdao University, Qingdao, Shandong Province 266003, China; ^4^Traditional Chinese Medicine Preparation, The Second Affiliated Hospital of Xi'an Jiaotong University, Xi'an, Shanxi Province 710004, China

## Abstract

*Purpose*. To evaluate visual outcomes and identify prognostic factors after pars plana vitrectomy (PPV) surgery for traumatic endophthalmitis.* Methods*. Medical records of 121 consecutive patients (121 eyes) diagnosed with traumatic endophthalmitis that had undergone pars plana vitrectomy were retrospectively reviewed.* Results*. 121 patients, aged from 6 to 71 years, all underwent PPV surgery. 113 cases had improved best corrected visual acuity (BCVA) after surgery and 60% of them obtained BCVA better than fingers counting (FC). Good final visual prognosis was significantly associated with time between trauma and initial treatment less than 12 hrs (40% versus 98%; *P* < 0.001), time between trauma and PPV treatment less than 24 hrs (62% versus 98%; *P* < 0.001), laceration length less than 10 mm (63% versus 96%; *P* < 0.001), and presenting VA better than LP (42% versus 96%; *P* < 0.001), while gender, type of laceration, presence of IOFB or retinal detachment, and the use of silicone oil tamponade were not significant factors resulting in better BCVA. Bacteria were identified in 43.8% of specimens and most of the microorganisms were identified as nonvirulent ones.* Conclusions*. Pars plana vitrectomy surgery was preferred as a primary treatment option for traumatic endophthalmitis. A good final visual prognosis was significantly associated with timely treatment, prompt vitrectomy surgery, shorter length of laceration, and better presenting visual acuity.

## 1. Introduction

Traumatic endophthalmitis is a subset urgent and severe ophthalmologic disease, remaining as a notable cause of poor visual outcome. The type of pathogenic microorganism, nature of the injury, the presence of a foreign body, and the geographical region in which the trauma occurred are all important factors influencing both treatment and prognosis. Pars plana vitrectomy (PPV) treatment for traumatic endophthalmitis is an effective method. The roles of vitrectomy are multiple: it eliminates a sizeable portion of germs, toxins, and inflammatory cells; it clears the media; it eliminates the vitreous scaffolding that causes traction and subsequent retinal detachment. It directly determines the final visual prognosis of traumatized eyes [1–6]. In this respective review of treatment on traumatic endophthalmitis, we identify the susceptivity factors to obtain better postoperative visual acuity after PPV for the purpose of better reducing the incidence of vision impairment or blindness caused by traumatic endophthalmitis.

## 2. Patients and Methods

A detailed retrospective review was conducted on 121 patients (121 eyes) with penetrating ocular trauma and traumatic endophthalmitis, who presented to the Ophthalmology Department, the Affiliated Hospital of Qingdao University, in China from January 2004 to December 2008. The study protocol was approved by the Institutional Review Board of the Affiliated Hospital of Qingdao University. The diagnosis of traumatic endophthalmitis was made by the following: the new onset penetrating eyeball injury, excluding endogenous infectious history; severe loss of visual acuity and rapid deterioration to worse than 20/400; significant intraocular inflammation and hypopyon in anterior chamber and vitreous; intraocular hypopyon tested by vitreoretinal surgery; and positive culture results from vitreous cavity hypopyon. The clinical symptoms and signs of endophthalmitis included eye pain, decreased visual acuity, tearing, corneal edema, chemosis, conjunctival hyperaemia, corneal, limbus, or scleral perforation, reduced red reflex, and afferent pupillary defect.

121 patients, aged from 6 to 71 years, with mean 44.2 ± 5.9 years of age, all underwent pars plana vitrectomy (PPV). Among them, 53 patients had intraocular foreign body and 37 patients had retinal detachment. 24 patients had intraocular foreign body and retinal detachment simultaneously. Most of these cases were given silicone oil tamponade or lensectomy at the same time. In summary, 98 cases underwent silicone oil tamponade. 116 patients underwent lensectomy and only 5 patients had their lens kept. 27 patients underwent intraocular lens implantation. 85 cases had corneal perforation and 36 cases had scleral perforation (according to the longer perforation position occurring in cornea or sclera). All 121 eyes showed hypopyon. Vitreous cavity specimen was extracted during vitrectomy for bacterial and fungal cultures. Vancomycin was added to BSS infusion fluid as valid drug against Gram-positive microorganisms in the vitreous cavity during vitreoretinal surgery.

Patients demographic studies included age, sex, place of trauma, mechanism of injury, the location and size of corneal perforation or scleral perforation, with or without uveal prolapse, entry and location of IOFB, time between injury and repair, and initial and final best corrected visual acuity. The concurrent cataract, vitreous hemorrhage, hypopyon, retinal detachment, and culture results of isolated microorganisms were also examined. The clinical variable factors associated with postoperative visual acuity including gender, time between injuries and treatment or PPV surgery, laceration length, presenting visual acuity, with IOFB or not, retinal detachment, corneal or scleral perforation, lensectomy, and silicone oil tamponade treatment were specifically analyzed.

Statistical analysis was carried out using SPSS17.0 software (SPSS Inc., Chicago, IL, USA). Categorical variables were analyzed using *χ* ^2^ and Fisher's exact test. *P* < 0.05 was taken as a level of statistical significance.

## 3. Results

Of these 121 patients, 119 cases kept their globes and 2 cases underwent evisceration in the end because of recurrence endophthalmitis. Before timely surgery treatment, 74% of the patients suffered severe vision impairment and endured BCVA worse than fingers counting (FC). After emergent PPV surgery, 113 cases had increased best corrected visual acuity and 60% of them obtained BCVA better than FC (*χ* ^2^ = 100.98, *P* < 0.01). Also, 41 patients obtained BCVA better than 20/200, indicating the substantial reduction of the incidence of vision impairment or blindness after vitreoretinal surgery for traumatic endophthalmitis patients (Table 1).

Four clinic factors associated with better post-op BCVA were identified by univariate analysis (Table 2). Time between trauma and initial treatment less than 12 hrs, time between trauma and PPV treatment less than 24 hrs, laceration length less than 10 mm, and presenting VA better than LP were statistically significant factors which could result in better BCVA, while gender and the perforation position were not significant factors. More than 95% of the patients without IOFB or retinal detachment could get BCVA improvement just after PPV surgery. For those with IOFB or retinal detachment ones, 91 patients (92%) with silicone oil tamponade and 109 patients (93%) that underwent lensectomy recovered and obtained improved BCVA. The prognosis for mild traumatic endophthalmitis was favorable after PPV treatment. Those serious cases could also obtain better prognosis and benefit from PPV as well as the use of silicone oil or removing lens. The patients were followed up for 18–24 months, with a mean of 21.42 ± 2.17 months. 119 cases kept their globes and endophthalmitis was controlled almost entirely. Those cases kept stable intraocular structure and retinal detachment after removal of silicone oil at the end of the follow-up.

53 specimens (from 53 eyes) were cultured positively, mostly Gram-positive microorganisms (Table 3). The results included* Staphylococcus epidermidis*, the most common one, in 22 eyes (41%),* Staphylococcus aureus* in 12 eyes (22%), and* Bacillus cereus* in 7 eyes (13%). Others included* Escherichia coli* (2 cases),* Staphylococcus saprophyticus* (2 cases),* Acinetobacter* (1 case),* Bacillus subtilis* (1 case),* Pseudomonas aeruginosa* (1 case), and* Proteus* (1 case). Fungus infections including* Aspergillus fumigatus* (1 case),* Fusarium* (1 case), yeast like fungi (1 case), and* Candida albicans* (1 case) were also cultured positively.

## 4. Discussion

The incidence of traumatic endophthalmitis ranges from 6.8% to 9.5%, remaining as one of the leading causes of noncongenital unilateral blindness [7]. Risk factors for traumatic endophthalmitis include presence of an IOFB, injury in a rural setting, wound contamination with organic matter, primary wound closure delayed for longer than 24 h after injury, and involvement of the lens capsule [8–11]. Vitrectomy treatment is suggested to be performed immediately after endophthalmitis is diagnosed [12–14]. In our cases, results also firmly evidenced that the immediate vitrectomy for traumatic endophthalmitis with suitable intravenous medication was effective to preserve the eyeball and visual acuity. Of all 121 patients, 98% (119/121) of the patients kept their globes, 93% (113/121) of the patients achieved increased best corrected visual acuity after vitrectomy, and 98% (103/105) of the patients that received PPV within 24 hours obtained increased BCVA. Besides, 34% of the patients (41/113) got BCVA better than 20/200. There are still 3 patients who even got BCVA better than 20/25. Endophthalmitis was much alleviated after the initial emergent vitrectomy and was brought under control with the continued usage of intravenous and topical antibiotics. The possible mechanism was that early emergent vitrectomy could remove the infecting microbiologic load and allow better diffusion of antibiotics within the eye, which might be helpful in retaining useful vision.

Previous studies have demonstrated that the clinical features associated with better visual acuity outcomes included better presenting visual acuity, early presentation to the institutes, and isolation of a nonvirulent organism [15, 16]. In the current study, similar results were demonstrated. 96% of the patients who presented with visual acuity better than LP, 96% of the patients who got laceration of less than 10 mm, 98% of the patients who received initial treatment of less than 12 hrs, and 98% of the patients who were treated by PPV surgery after trauma of less than 24 hrs all received increased BCVA. Time between trauma and initial treatment less than 12 hrs, time between trauma and PPV treatment less than 24 hrs, laceration length less than 10 mm, and presenting VA better than LP were statistically significant factors which could result in better visual prognosis.

For those patients with severe vitritis, more than 90% of the cases also obtained BCVA improvement after PPV and silicone oil tamponade or lensectomy simultaneously. The usage of silicone oil tamponade was determined by the degree of severity of endophthalmitis in the present study. 98 patients with retinal detachment, huge and multiple retina holes, retina degeneration, or proliferative vitreoretinopathy underwent PPV and silicone oil tamponade simultaneously. As for the use of silicone oil in traumatic eyes, the main indication for silicone oils is complicated retinal detachment. Azen et al. [17] reported complete reattachment rates of 62% for traumatic detachment and maculae attachment in 88% of the traumatic patients, while, in other cases without retinal detachment, the silicone oil tamponade also plays an important role in preventing the development of proliferative vitreoretinopathy (PVR) and extensive scarring process [18, 19]. Theories demonstrated that, in the first phase of traumatic injuries, vitrectomy could prevent infection or inflammation, while, in the second phase of the wound healing process, silicone oil tamponade could inhibit the following cell proliferation. In the present study, 92% of the patients obtained BCVA improvement after PPV and silicone oil tamponade simultaneously, which was the same result as mentioned above. Thus, if the severe vitritis is definite, vitrectomy with silicone oil tamponade treatment could be administered promptly as an appropriate and as the most effective way to control inflammation.

In our study, there were actually no differences in the visual acuity prognosis between traumatized eyes with and without the use of silicone oil or lensectomy. Different severity of traumatic endophthalmitis determined different choice of treatment. In this study, the silicone oil tamponade or lensectomy was used for those with serious traumatic endophthalmitis, while patients with mild vitritis could benefit from prompt PPV only without the use of silicone oil or lensectomy. That is probably the reason why the use of silicone oil or lensectomy was not a significant factor associated with better post-op BCVA. Actually, the surgical exploration and detailed ocular injury evaluation should be performed carefully before PPV or other kinds of treatment. The selection of treatment methods for traumatic endophthalmitis was mostly determined by the severity degree of ocular trauma and posttraumatic endophthalmitis. The endophthalmitis itself and the corresponding treatment directly affect the prognosis of traumatized eyes.

Lieb et al. [20] classified the cultured organisms as follows: coagulase-negative* Staphylococci*,* Corynebacterium*, and* Propionibacterium acnes* were nonvirulent organisms, whereas all other organisms were virulent organisms. One of the clinical features associated with better visual acuity outcomes was culture of nonvirulent organism, while the recurrence of endophthalmitis is the key to the loss of the eyeball which might be infected by virulent microorganisms. In the present study, the most frequent infected microorganism was* Staphylococcus epidermidis* accounting for almost 41% (22/53) of all patients, which was in agreement with previous reports [6]. Additionally, the results that most infected microorganisms were identified as nonvirulent ones might be one of the reasons why those satisfactory visual outcomes could be obtained in this retrospective review.

In conclusion, prompt PPV is an effective treatment for traumatic endophthalmitis patients after the diagnosis is determined. Besides, the better visual acuity prognosis postoperatively could benefit from prompt vitrectomy treatment, isolation of nonvirulent microorganisms, shorter length of laceration, better presenting visual acuity, and the use of silicone oil or removing the lens if necessary.

## Figures and Tables

**Table 1:** BCVA outcome pre/postoperatively.

Visual acuity	Preoperatively (%)	Postoperatively (%)
NLP	7 (5.79)	5 (4.13)
LP	35 (28.93)	5 (4.13)
HM	48 (39.67)	16 (13.22)
FC	27 (22.31)	23 (19.01)
⩽20/400	4 (3.31)	31 (25.62)
20/200~20/50	0	32 (26.45)
20/50~20/40	0	6 (4.96)
*⩾*20/25	0	3 (2.48)

NLP: no light perception; LP: light perception; HM: hand motion; FC: fingers counting.

**Table 2:** Presentation features associated with better BCVA.

Factor	Number	Improved BCVA (%)	*P* value
Gender (*n* = 121)			
Male	103	98/103 (95.15)	0.1781
Female	18	15/18 (83.3)	

Treatment period (*n* = 121)			
<12 hrs	111	109/111 (98.20)	0.0000
>12 hrs	10	4/10 (40)	

PPV period (*n* = 121)			
<24 hrs	105	103/105 (98.10)	0.0000
>24 hrs	16	10/16 (62.5)	

Laceration length (*n* = 121)			
⩽10 mm	110	106/110 (96.36)	0.0004
>10 mm	11	7/11 (63.63)	

Presenting VA (*n* = 121)			
*⩾*LP	114	110/114 (96.49)	0.0000
<LP	7	3/7 (42.86)	

IOFB			
Yes	53	48/53 (90.57)	0.4627
No	68	65/68 (95.59)	

Retinal detachment			
Yes	37	33/37 (89.19)	0.4028
No	84	80/84 (95.24)	

Corneal perforation			
Yes	85	82/85 (96.47)	0.0898
No	36	31/36 (86.11)	

Scleral perforation			
Yes	36	31/36 (86.11)	0.0898
No	85	82/85 (96.47)	

SO use			
Yes	98	91/98 (92.86)	0.9846
No	23	22/23 (95.65)	

Lensectomy			
Yes	116	109/116 (93.97)	0.7555
No	5	4/5 (80)	

PPV: pars plana vitrectomy; IOFB: intraocular foreign body; SO: silicone oil.

**Table 3:** Summary of culture organisms.

Culture-proven microorganisms	Eyes	Percentages (%)
*Staphylococcus epidermidis*	22	41.5
*Staphylococcus aureus*	12	22.6
*Bacillus cereus*	7	13.2
Fungus	4	7.5
Other microorganisms	8	15.1
